# Accuracy of remote, video-based supraventricular tachycardia detection in patients undergoing elective electrical cardioversion: a prospective cohort

**DOI:** 10.1007/s10877-025-01263-5

**Published:** 2025-01-29

**Authors:** Iris Cramer, Rik van Esch, Cindy Verstappen, Carla Kloeze, Bas van Bussel, Sander Stuijk, Jan Bergmans, Marcel van ’t Veer, Svitlana Zinger, Leon Montenij, R. Arthur Bouwman, Lukas Dekker

**Affiliations:** 1 Department of Electrical Engineering, Eindhoven University of Technology, Groene Loper 3, 5612 AZ Eindhoven, the Netherlands; 2 Department of Anesthesiology, Intensive Care and Pain Medicine, Catharina Hospital, Eindhoven, the Netherlands; 3 Department of Cardiology, Catharina Hospital Eindhoven, Eindhoven, the Netherlands; 4 Department of Medical Physics, Catharina Hospital, Eindhoven, the Netherlands; 5 Department of Intensive Care Medicine, Maastricht University Medical Centre+, Maastricht, the Netherlands; 6 Care and Public Health Research Institute CAPHRI, Maastricht University, Maastricht, the Netherlands; 7 Cardiovascular Research Institute Maastricht CARIM, Maastricht University, Maastricht, the Netherlands

**Keywords:** Physiologic monitoring, Remote photoplethysmography, Supraventricular tachycardia, Video-camera

## Abstract

Unobtrusive pulse rate monitoring by continuous video recording, based on remote photoplethysmography (rPPG), might enable early detection of perioperative arrhythmias in general ward patients. However, the accuracy of an rPPG-based machine learning model to monitor the pulse rate during sinus rhythm and arrhythmias is unknown. We conducted a prospective, observational diagnostic study in a cohort with a high prevalence of arrhythmias (patients undergoing elective electrical cardioversion). Pulse rate was assessed with rPPG via a visible light camera and ECG as reference, before and after cardioversion. A cardiologist categorized ECGs into normal sinus rhythm or arrhythmias requiring further investigation. A supervised machine learning model (support vector machine with Gaussian kernel) was trained using rPPG signal features from 60-s intervals and validated via leave-one-subject-out. Pulse rate measurement performance was evaluated with Bland–Altman analysis. Of 72 patients screened, 51 patients were included in the analyses, including 444 60-s intervals with normal sinus rhythm and 1130 60-s intervals of clinically relevant arrhythmias. The model showed robust discrimination (AUC 0.95 [0.93–0.96]) and good calibration. For pulse rate measurement, the bias and limits of agreement for sinus rhythm were 1.21 [− 8.60 to 11.02], while for arrhythmia, they were − 7.45 [− 35.75 to 20.86]. The machine learning model accurately identified sinus rhythm and arrhythmias using rPPG in real-world conditions. Heart rate underestimation during arrhythmias highlights the need for optimization.

**Supplementary Information:**

The online version contains supplementary material available at 10.1007/s10877-025-01263-5.

## Introduction

Early identification of postoperative complications continues to be a critical issue within perioperative medicine [1, 2]. Incidences of new-onset atrial fibrillation among ward patients undergoing surgery can reach up to 20%, for example after esophagectomy while escalating further, ranging from 7.9 to 37.6%, following cardiac surgery [1, 3, 4]. The current approach to recognize postoperative complications on general wards in hospitals wards has several limitations. First, this practice is time consuming, as it is based on intermittent vital sign measurements by health care workers, and protocol adherence thereby is low, which drives incomplete and missing values [5, 6]. Moreover, intervals between checks are typically within an order of 8 h, which is long as vital signs deviations may occur suddenly and often occur several hours before an adverse event [7]. Furthermore, current early warning protocols are generic, and population differences may influence efficacy [2].

Continuous pulse rate monitoring using video, with sensitive arrhythmia detection embedded in prediction algorithms, could potentially improve detection of clinical deterioration. Notably, new-onset atrial fibrillation is an important predictor of various other complications [1]. In high-care departments, multiple-lead ECG monitoring is used for arrhythmia detection, but this technology is not employed on general wards due to the obtrusiveness and associated workload. This workload could be alleviated through implementation of novel monitoring technologies, for instance video-based monitoring technology [8, 9]. Video-based heart rate measurement can be performed using remote photoplethysmography (rPPG) [10] estimating heart rate using subtle changes in skin color, caused by the cardiovascular pulse wave. Machine learning models can integrate these highly granular rPPG data to inform physicians. Video monitoring offers patients the advantage of retaining their freedom of movement, as they are not constrained by equipment or mandated to wear sensors. Another advantage of video technology is that it can also measure various other vital sign and context, for example seizures, pneumothorax, or specific facial cues [11–14].

While a few studies have shown the feasibility of video-based arrhythmia detection in controlled settings [15–17], only brief time windows were used if the patients’ face remained unmoved, which limits application for continuous monitoring in clinical settings. Before implementing such a technique in clinical practice, it is important to assess its performance under real-world, clinical conditions, where patient motion is allowed. Additionally, it is of interest to be able to detect other arrhythmias alongside atrial fibrillation and to accurately measure heart rate during arrhythmias for rate control strategies. Furthermore, in previous research high-resolution cameras were used [15], which limits scalability due to the high costs of these cameras.

The primary objective was to determine the performance of rPPG-based pulse rate estimation in a clinical setting, using low-cost, off-the-shelf cameras, combined with an arrhythmia machine learning algorithm for the detection of supraventricular arrhythmias. Therefore, we hypothesized that the pulse rate measurements in both sinus rhythm and cardiac arrhythmias by rPPG are accurate compared to ECG monitoring. Next, we hypothesized that a supervised machine learning model for highly granular data has good diagnostic discrimination and calibration to detect irregular heart rate in a prospective cohort of patients who underwent cardioversion. During the study, this hypothesis was expanded to encompass not only the detection of irregular heart rates, but all clinically relevant arrhythmias identified in the cohort.

## Methods

### Study design

A prospective, observational diagnostic study was performed among patients with atrial fibrillation or atrial flutter who were admitted for elective, electrical cardioversion in a tertiary care hospital (Catharina hospital, Eindhoven, the Netherlands) (Appendix A: STARD checklist). According to the Dutch directives on research in human subjects, the study was reviewed by the medical ethical committee of the Maxima Medical Centre (Eindhoven, the Netherlands, File no: N21.039). The study protocol was approved by the internal review boards of Catharina hospital Eindhoven. The study was registered in the Dutch Trial Register with number NL9854 (please see Overview of Medical Research in the Netherlands at https://onderzoekmetmensen.nl/en/trial/25838).

### Study population and procedures

Patients eligible for inclusion were adults (≥ 18 years) diagnosed with atrial fibrillation or atrial flutter, who were planned for elective cardioversion at the Emergency Cardiac Care unit of Catharina hospital Eindhoven between August and November 2021. Patients were excluded if they had a pacemaker-dependent rhythm. Due to logistical study reasons one patient could be included per half-day. Written informed consent was obtained prior to starting the research procedures. Sample size was calculated for the comparison of the area under a ROC curve with a null hypothesis value (α = 0.05, β = 0.20, AUC = 0.725, null hypothesis value of 0.5 and a ratio of negative/positive groups of 1). This resulted in a sample size of 48. To account for the expectation that approximately 15% of patients would not convert to sinus rhythm, which would lead to a drop out from the analysis [18], the total sample size was increased to 56. Continuous video-recordings and single-lead ECG as reference standard were simultaneously collected at least 10 min before, during and 10 min after the cardioversion. The exact duration varied depending on the clinical situation. During data collection, patients were confined to their beds, without restricting their range of movement. Time periods during which the patients’ face was covered because of mask ventilation or because healthcare providers were working in front of the patient were annotated manually in the videos to exclude those from further analysis.

### Investigational setup

Patients were monitored with a video camera mounted on a specifically developed medical-grade trolley (iTD Pro-cart), positioned at the foot-end of the patients’ bed, approximately 2 m from the patient’s face. The videos were obtained with a single Red Green Blue camera (UI-3860CP-C-HQ rev. 2, IDS Imaging Development Systems GmbH, Obersulm, Germany) with a frame rate of 32 Hz and spatial resolution of 484 × 274 pixels. The acquisition was performed on a laptop (HP) with Visual Studio 2017 (Microsoft). Heart rate was derived from single-lead ECG (IntelliVue MP70 patient monitor (Philips, Eindhoven, the Netherlands), 500 Hz) with Intellivue Datalogger software.

### Data preprocessing

Post-processing of video data was done using Matlab 2021b (MathWorks, USA). After excluding face-covered video data due to masks or health care providers, the rPPG waveform was retrieved using the Plane-Orthogonal-to-Skin method of Wang et al. [10, 19]. The rPPG waveform was further processed to detect the peak, which corresponds to a heartbeat, using the findpeaks function in Matlab (Appendix B). R-peaks of ECG data were detected with automated R-peak detection software (R-DECO, Version 3, 29 June 2007) and subsequently verified for accuracy by a clinical expert. Then, rPPG and ECG were synchronized to ensure alignment of their timestamps. Multiple 60-s intervals per patient were created.

### Data analysis

First, ECG data was diagnosed into two classes by a cardiologist: (1) normal sinus rhythm and (2) arrhythmia requiring further clinical investigation. Then, the supervised machine learning model was trained using support vector machine with a Gaussian kernel, rPPG signal features were used as input variables and described in detail elsewhere [26]. No correction for repeated measures was deemed necessary due to the consistent number of measurements per patient. Due to the relatively small study sample size, leave-one-subject-out validation was chosen to ensure that data from the same patient did not appear in both the training and the test set. Discrimination of the model was assessed using an ROC curve where confidence intervals were calculated via bootstrapping. The operating point of the curve was chosen using the Youden index to account for imbalance in the dataset [27]. The calibration of the model was assessed graphically using a calibration plot.

We investigated the reliability of pulse rate measurements based on rPPG in comparison to the reference ECG. Median heart rate over 60-s intervals from rPPG and reference ECG were compared, and absolute errors were calculated. Subsequently, agreement between rPPG and ECG was visualized by Bland Altman plots [20] and scatter plots. We verified whether the differences between the measurements followed a normal distribution and whether the variability of these differences remained consistent. Considering multiple measurements per patient (paired observations), the limits of agreement and their confidence intervals were calculated considering both within- and between-patient variability using the modified Bland–Altman method for repeated measurements described by Zou, and the confidence intervals around the limits of agreement values were estimated using the method of variance estimate recovery (MOVER) [21]. In addition, the Pearson correlation was calculated, and intra-class correlation was determined using two-way mixed-effects models [22]. The heart rate evaluation was compared to the reference standard, which requires an accuracy within –5 bpm to 5 bpm or –10 to 10% (whichever is larger) [23]. Data analysis was performed using RStudio (version 2022.12.0).

## Results

### Population

**Fig. 1:**
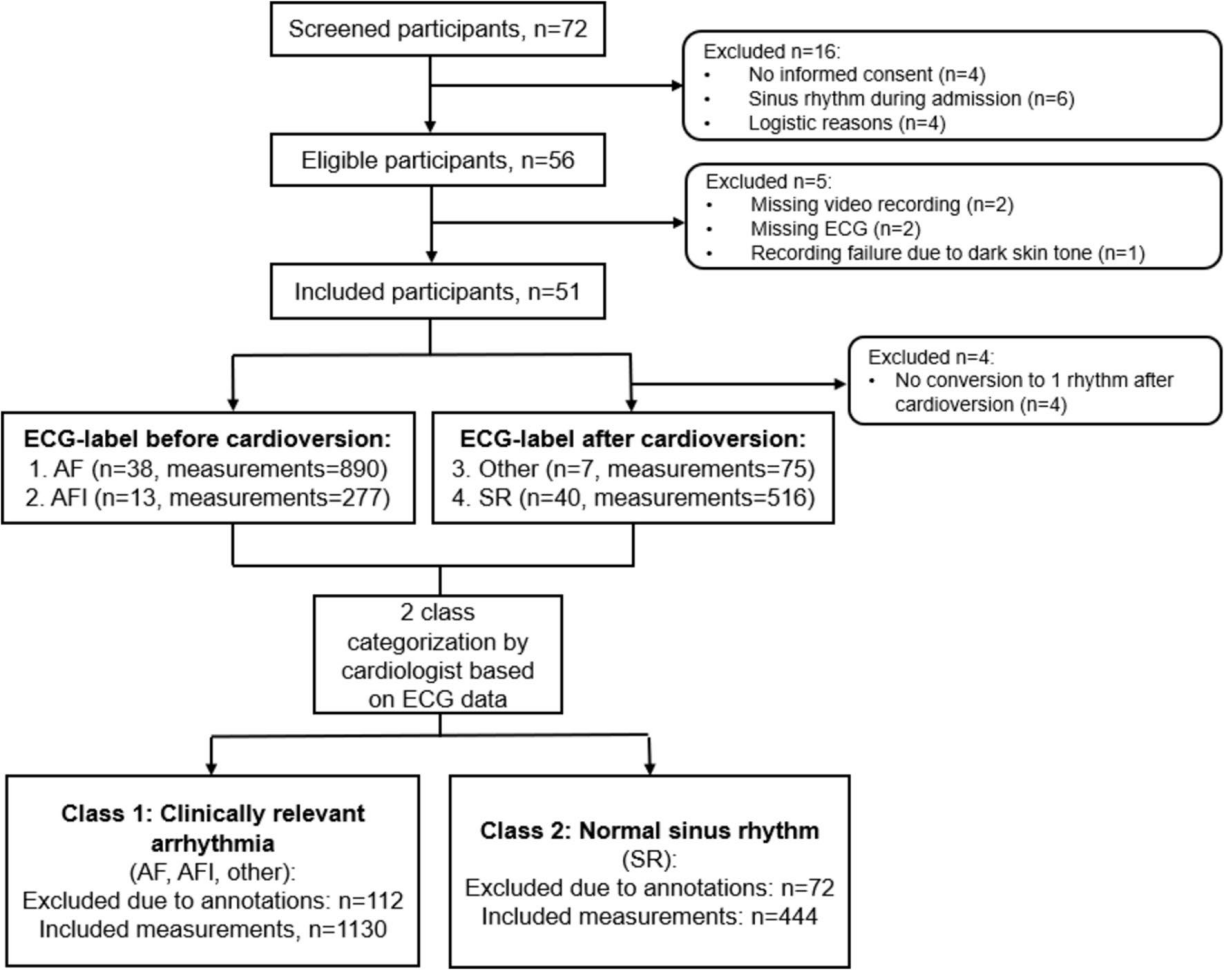
Study flowchart

Out of a screened total of 72 patients, 56 patients were enrolled in the study (Fig. 1, Table 1). Five patients had to be excluded. Two had recording failure due to missing video recordings; two had missing reference data; and one was excluded because the initial prototype and algorithms were not tailored for high quality beat detection in individuals with a darker skin tone. This resulted in 51 patients with an average recording duration of 40 (± 17 SD) minutes per patient. Before treatment, ECGs of 38 patients were classified as atrial fibrillation and of 13 patients as atrial flutter by a cardiologist. After treatment, 40 ECGs were classified as sinus rhythm and 7 as “other arrhythmia” (one with first-degree atrioventricular block, one with third-degree atrioventricular-block, one with sinoatrial block, three with atrial tachycardia, and one with sinus rhythm with presumed clinically relevant premature complexes). Furthermore, the post-treatment segments of 4 patients were excluded, due to the absence of a definable class by the cardiologist resulting from variable rhythms (no conversion to 1 rhythm after treatment). For arrhythmia and sinus rhythm, 9.9% and 15.9% of the recording time were excluded, because clinical interventions had been annotated.

**Table 1:** Patient characteristics

Patient characteristics (N = 51)	
Sex, n. (%)	
Female	18 (35.3)
Male	33 (64.7)
Age, yr, median, IQR	69 (11.5)
BMI, kg/m^2^, mean, SD	28.31 (4.2)
Skin type, n. (%)	
Fitzpatrick I-II	50 (98.0)
Fitzpatrick III-IV	1 (2.0)
Comorbidities, n. (%)	
Hypertension	23 (45.1)
Heart failure	9 (17.4)
Valve disease	2 (3.9)
Diabetes Mellitus	7 (13.7)
Asthma/COPD	5 (9.8)
Malignancy	4 (7.8)

### Arrhythmia detection

**Fig. 2:**
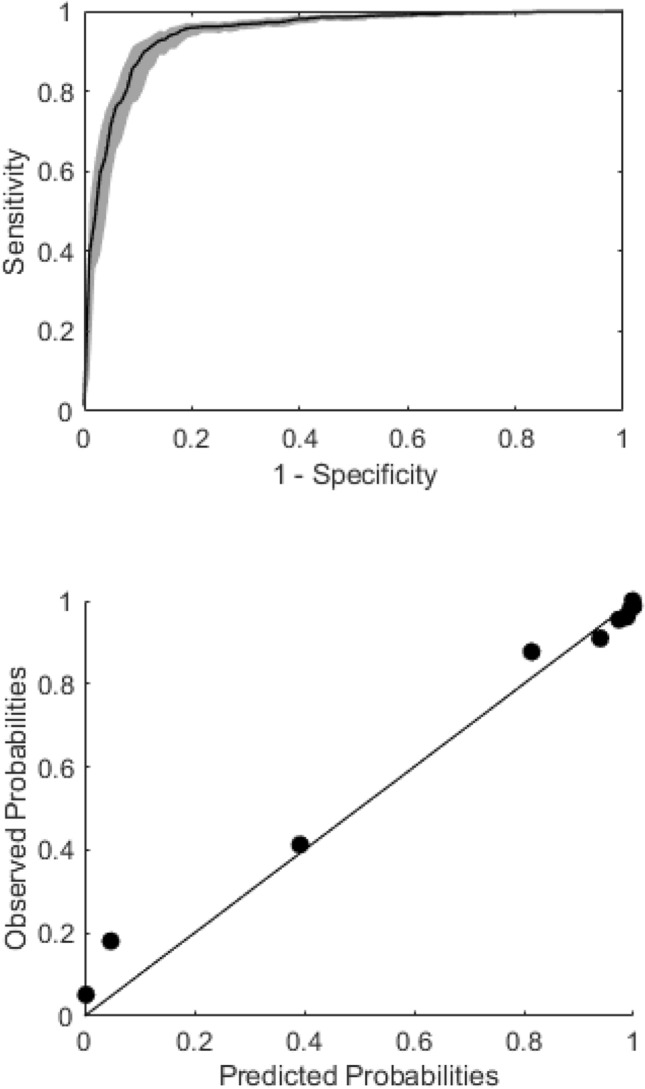
**A** Discrimination with AUC: 0.95 [0.93–0.97]. **B** Calibration curve

Discrimination and calibration of the machine learning model were good (Tables 2, 3, Fig. 2A, B) with an AUC for supraventricular arrhythmia classification of 0.95 [0.93–0.97].

**Table 2:** Cross tabulation of the rPPG algorithm vs ECG

Arrhythmia rPPG	Rhythm class ECG				Total
	AF	AFl	Other	SR	
Positive	766	233	42	54	1095
Negative	44	19	26	362	451
Total	810	252	68	416	1546

**Table 3:** Diagnostic accuracy of the rPPG algorithm

	60 s intervals
AUC	0.95 [0.93–0.96]
Sensitivity (%)	92.1
Specificity (%)	87.0
PPV (%)	95.1
NPV (%)	80.3

*AUC* area under the curve, *NPV* negative predictive value, *PPV* positive predictive value

Misclassification of the atrial fibrillation patients was as follows; 5.4% of the 60-s intervals were wrongly misclassified as normal sinus rhythm. These errors mainly occurred in segments of 2 patients with atrial fibrillation with a low ventricular response (< 40/min) (55% of the incorrectly classified atrial fibrillation segments). The other errors occurred in patients with a ventricular response of 40–60/min. Of the atrial flutter cases, 7.5% of the 60-s intervals were wrongly classified as normal sinus rhythm. In most cases, ventricular response was slower than 70/min. Within the "other arrhythmia" group, 38,2% of the segments were inaccurately categorized as normal sinus rhythm. These errors mainly occurred in 60-s intervals of patients with a first- and third-degree atrioventricular-block and sinoatrial-block. Within the “normal sinus rhythm” group, 13,0% of the segments were incorrectly classified as arrhythmia. In most cases, these 60-s intervals presented multiform premature atrial complexes and premature ventricular complexes thereby creating an irregular-appearing rhythm.

### Reliability of rPPG-based heart rate measurements

**Fig. 3:**
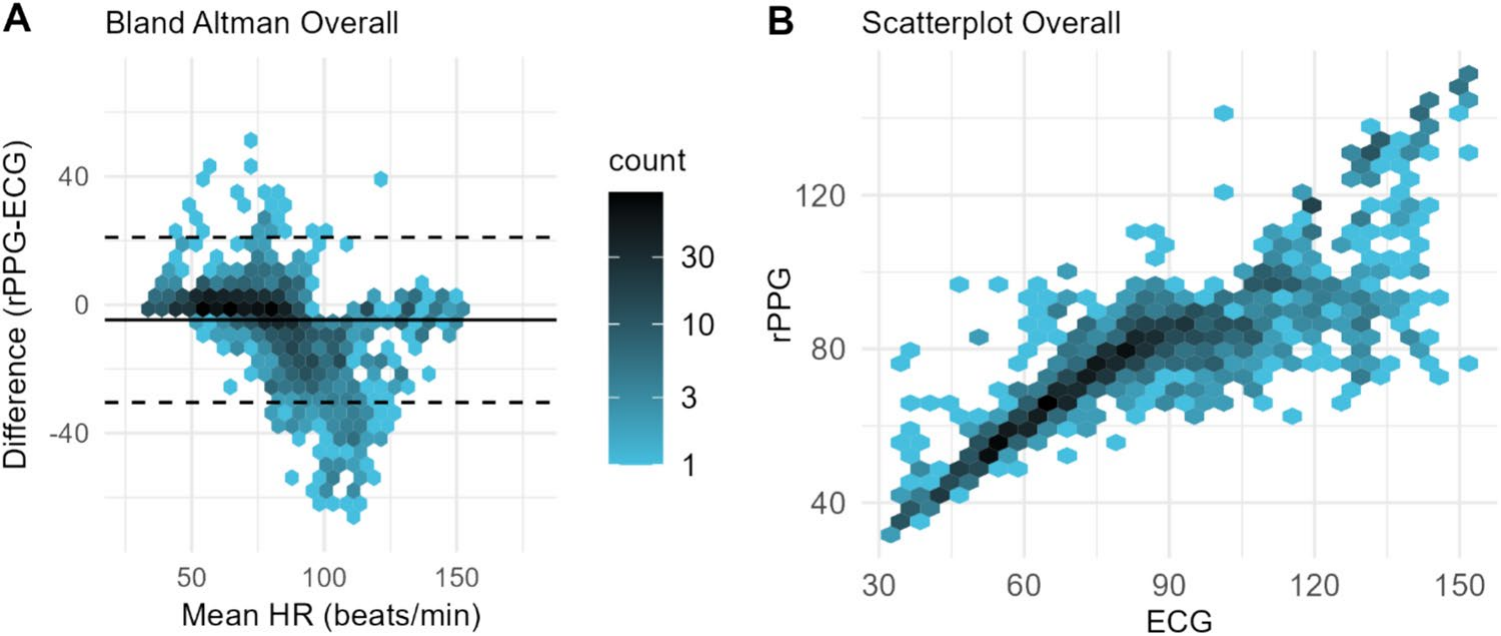
**A** Representation depicting the overall agreement between rPPG and ECG, where the difference of between rPPG and ECG is plotted on the y-axis, and the mean heart rate is plotted on the x-axis. Each data point represents a 60-s interval. Bias is indicated by the black lines and limits of agreement are indicated by the dotted lines. **B** A scatterplot illustrating the relationship between heart rates measured by ECG (x-axis) and rPPG (y-axis). Each data point represents a 60-s interval. Points closely aligned with the diagonal line indicate strong agreement between the two measurement methods, while deviations from this line reflect discrepancies between rPPG and ECG readings

**Fig. 4:**
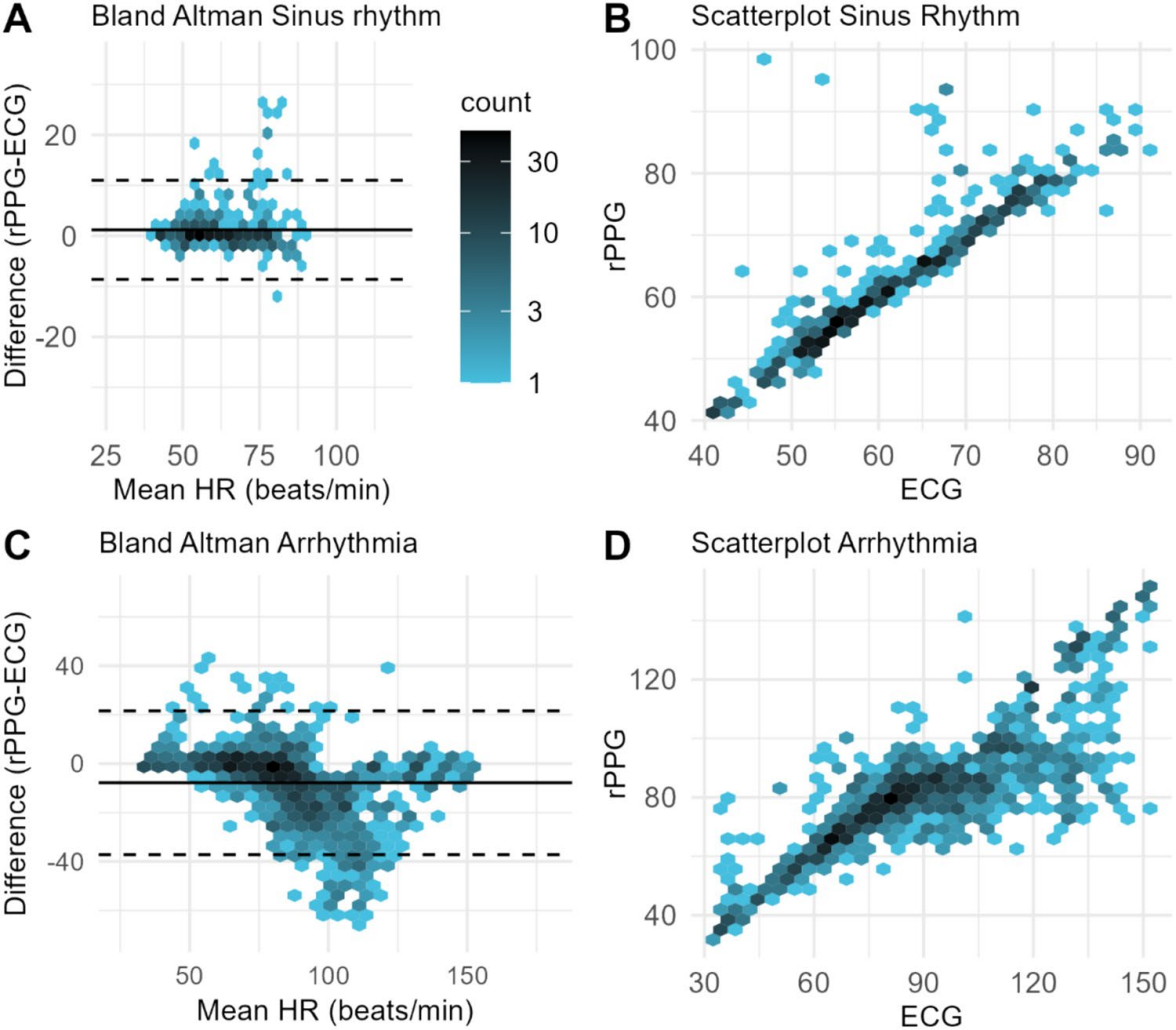
**A**, **C** Representation depicting the agreement between rPPG and ECG, where the difference of between rPPG and ECG is plotted on the y-axis, and the mean heart rate is plotted on the x-axis. Each data point represents a 60-s interval. Bias is indicated by the black lines and limits of agreement are indicated by the dotted lines. In figure **C**, the mean and limits of agreement are provided as a reference, but it should be noted that these values may not be entirely reliable due to the presence of heteroscedasticity. **B**, **D** Scatterplots illustrating the relationship between heart rates measured by ECG (x-axis) and rPPG (y-axis). Each data point represents a 60-s interval. Points closely aligned with the diagonal line indicate strong agreement between the two measurement methods, while deviations from this line reflect discrepancies between rPPG and ECG readings

The overall agreement between rPPG and ECG was shown in Fig. 3A, B and Table 4. For sinus rhythm, 90.8% of the measurements differed ≤ 5 bpm from the ECG measurements (Table 4, Fig. 4A, B). For arrhythmia, 48.6% of the measurements differed ≤ 5 bpm from the measurements of ECG (Table 4, Fig. 4C, D). In addition, for atrial fibrillation, 8 patients had a mean percentage error of more than −10% (Appendix C). For atrial flutter, 5 patients had a mean percentage error of more than −10% and 1 patient of + 10%. For the other arrhythmia group, 2 patients had a mean percentage error of more than −10% and 2 patients of + 10%. Results of the Error Grid analyses show that most of the measurements lie in zone A, whereas Zone B includes almost all the remaining measurements (Table 4, Fig. 4C, D).

**Table 4:** Agreement of rPPG compared with the ECG reference device

	Sinus Rhythm	Arrhythmia	Overall
Bland Altman			
Bias (bpm), (SE)	1.21 (5.00)	−7.45 (1.73)	−4.70 (1.22)
LLoA (bpm), 95% CI	−8.60 (7.26/10.35)	−35.75 (−41.92/−31.03)	−30.41 (−34.22/−27.40)
ULoA (bpm), 95% CI	11.02 (9.67/12.76)	20.86 (16.14/27.03)	21.02 (18.01/24.83)
Within 5 bpm, n (%)	403 (90.77)	578 (48.61)	981 (60.07)
Within 10%, n (%)	413 (93.02)	742 (62.41)	1155 (70.72)
r, 95% CI	0.90 (0.86/0.94)	0.82 (0.80/0.86)	0.86 (0.84/0.89)
ICC, 95% CI	0.84 (0.73/0.88)	–	–

*CI* confidence interval, *ICC* intraclass correlation, *LLOA* lower limit of agreement, *r* Pearson correlation, *SE* standard error, *ULoA* upper limit of agreement. The ICC was not calculated for arrhythmia and the overall dataset due to the presence of heteroscedasticity

## Discussion

The present prospective cohort study, comprising 51 patients, aimed to investigate arrhythmia and pulse rate detection using rPPG and ECG as reference has two main findings. First, rPPG-based discrimination of normal sinus rhythm versus clinically relevant arrhythmias was good, with an AUC of 0.95 under real-life, continuous conditions, while calibration was good as well. Second, rPPG underestimated heart rate during cardiac arrhythmias.

Other studies on arrhythmia detection with rPPG did not focus on allowing patient motion and employing low-resolution cameras. In contrast, our research demonstrated that by addressing these factors—allowing patient motion and employing low-resolution cameras—the diagnostic accuracy of our rPPG-based arrhythmia detection model remained consistent with the accuracy reported in other studies. Our reported discrimination level was consistent with findings in other studies [15, 17]. However, these studies did not report on the calibration of the models, which is essential for a comprehensive evaluation of model performance. Both studies were conducted in Asian populations [15, 17], whereas our study was conducted in a European population. These diverse settings suggest promising potential for the generalizability of our findings across different populations in the future. In addition, the above studies utilized deep learning [15, 17], whereas we employed supervised machine learning. An advantage of our approach is that supervised machine learning models can be more interpretable and require less computational power, making them potentially more practical for real-time clinical applications. Reported reasons of misclassification were multiple premature complexes and motion artefacts in sinus rhythm and low ventricular response in atrial fibrillation, which is in line with our results [17, 28].

Our sinus rhythm measurements show better agreement and coverage with the reference signal than prior studies [12, 16, 29]. Previous work in intensive care patients after cardiac surgery showed a bias of −3.7 ± 16 bpm during sinus rhythm compared to an ECG reference [29]. Pulse rate was detected within 5 bpm margins in 83% of total valid recording time. Another study in intensive care patients showed a bias of −2.0 with 95% limits of agreement of −6.4 and 2.5 beats/minute [12], with a total data loss was 46.8% of valid recording time. Our results of pulse rate measurement during arrhythmia show an underestimation of heart rate, which is line with the results of Couderc et al., they reported 17.7% of undetected cardiac beats [16].

For an effective early detection of arrhythmias and related complications in general hospitals wards, minimizing false negatives of the arrhythmia model is imperative. Therefore, we intentionally developed the model in a high-prevalence population which might inflate sensitivity achieving high-sensitivity monitoring. Although the prevalence of arrhythmias will be lower in a general ward setting, which may result in an increase in false positives [30], we need further investigation if this is similarly applicable to the general ward. Our main purpose is to accurately exclude arrhythmias in a general ward setting, thereby accepting a slightly higher number of false positives. Furthermore, rPPG can potentially serve as a tool to differentiate between patient groups requiring additional ECG monitoring and those who do not. The advantages include a more targeted focus on patients with rhythm disorders, providing more comfort for patients without rhythm disorders and potentially reducing costs. On the other hand, rPPG based pulse rate monitoring is less reliable during arrhythmias, which also underscores the importance of clinical judgment of the arrhythmia when this occurs, hence using the algorithm in a hospital setting.

This study has several limitations. Firstly, the quality of the rPPG signal of the only study patient with a dark skin tone (Fitzpatrick VI) was of insufficient quality to include in the analysis, due the fact that the initial prototype and algorithms were not tailored for high quality beat detection in individuals with a dark skin tone. Higher melanin concentration in a dark skin increases light absorption, which results is a weaker rPPG signal [31]. Due to the presence of only one patient in the dataset with a darker skin tone, it was not feasible to optimize the settings to ensure accurate functionality. However, a recent study shows that good performance can be achieved in patients with a dark skin tone using a light source of at least 100 lx or infrared light [32]. This infrared light source should be incorporated into future camera set-ups, because crucial to the advancement of video technology and algorithms is ensuring their inclusivity [13].

We opted to use 60-s medians in the present study. This option aligns with the intended clinical context of general ward monitoring, where the detection of sustained arrhythmias is prioritized over instantaneous beat-to-beat variability. However, our study population included patients with arrhythmia detection during electrical cardioversion. Here, shorter intervals might improve responsiveness. In contrast, using longer intervals helps to reduce the influence of transient noise artifacts, which may often occur with the sensitive rPPG. Taken together, shorter intervals more likely create more false positives in patient populations with lower incidence of arrhythmias than the patients in the present study, for example ward patients. The use of 60-s medians balances the applicability and technological feasibility for future ward patients.

In this study, an inherent imbalance was identified in the dataset, as the normal sinus rhythm group was smaller than the arrhythmia group. This imbalance could have notable implications for both the reliability and generalizability of our study findings. The decision not to address this imbalance by balancing the dataset was deliberate, as doing so would have incurred a substantial loss of valuable information related to the diverse range of arrhythmias present in our data. Each arrhythmia subtype contributes distinct characteristics and patterns. We aimed to mitigate the impact of the dataset imbalance on the assessment of model performance by incorporating the Youden Index into our analyses.

Future research should be directed towards the technical developments of new features to address current limitations of the model. Additionally, the model can be enhanced by incorporating various types of arrhythmias within a more extensive data set. Added value for early detection of deterioration can be achieved by also incorporating ventricular arrhythmias in the model. In addition, the results of arrhythmia detection and heart rate measurements can be improved with the development of a quality metric of the rPPG signal. This approach enables the automatic removal of measurements with poor signal quality, for example because of motion artefacts, which will lead to more accurate arrhythmia detection in clinical practice. Moreover, integration of other vital signs and context factors can further improve detection of deterioration in general ward patients [24, 25].

## Conclusion

The present study included a cohort with a high prevalence of arrhythmias, for which cardioversion was done. This allowed the study to demonstrate that a machine learning model could accurately differentiate normal sinus rhythm from clinically significant arrhythmias using rPPG under real-life, continuous monitoring conditions. This first step is encouraging to further investigate this technology in other settings, such as perioperative and general ward environment. While the lower prevalence of arrhythmias in these contexts may increase the risk of false positives, strategies such as using 60-s medians are expected to mitigate this issue. In addition, caution is advised when evaluating heart rate during arrhythmias, as there is potential for underestimation. Further research is needed to evaluate the feasibility and accuracy of this technology in perioperative and general ward applications.

## Supplementary Information

Below is the link to the electronic supplementary material.

Supplementary file1 (DOCX 419 KB)

## Data Availability

The datasets generated and analyzed during this study are not publicly available due to their sensitive and identifiable nature, as well as the restrictions imposed by the ethics protocol to safeguard the privacy of the subjects involved.
